# Case report: Posterior reversible encephalopathy syndrome, an adverse effect of lenvatinib and pembrolizumab combination therapy, in a patient with advanced endometrial cancer

**DOI:** 10.3389/fonc.2022.1079716

**Published:** 2023-01-20

**Authors:** Yuki Matsuura, Haruka Nishida, Takashi Kosaka, Kazuyuki Shigekawa, Kazuki Takasaki, Takayuki Ichinose, Mana Hirano, Haruko Hiraike, Kazunori Nagasaka

**Affiliations:** Department of Obstetrics and Gynecology, Teikyo University School of Medicine, Tokyo, Japan

**Keywords:** endometrial cancer, lenvatinib, pembrolizumab, posterior reversible encephalopathy syndrome, adverse effect

## Abstract

**Background:**

Lenvatinib-pembrolizumab combination (LEAP) is an approved therapy in Japan for advanced endometrial cancer, based on the data from the KEYNOTE-775 clinical trial. We report a case of posterior reversible encephalopathy syndrome (PRES) in a patient who received LEAP therapy for advanced endometrial cancer.

**Case presentation:**

A 53-year-old patient with stage IVB endometrial cancer having rectal metastases, after four cycles of paclitaxel-carboplatin therapy, was found to have increased rectal invasion, peritoneal dissemination, and multiple paraaortic lymph node metastases. She was treated with LEAP therapy and discharged on day 12 without adverse events, except for mild anemia on day 11 of treatment. She was carefully managed in the outpatient department, but on day 18, she was admitted to the emergency department with severely impaired consciousness and generalized seizures. Computed tomography of the head and lumbar tap showed no abnormal findings, and the seizures resolved with anticonvulsant medication alone. Based on a thorough physical examination and findings on magnetic resonance imaging (MRI), which showed high signal intensity in the left occipital lobe, encephalopathy, rather than encephalitis, was the likely diagnosis. Symptomatic improvement was observed, and pembrolizumab monotherapy was resumed.

**Conclusions:**

If consciousness is impaired during LEAP treatment, it is necessary to differentiate between immunogenic encephalitis caused by pembrolizumab or encephalopathy caused by lenvatinib. MRI and lumbar tap can help in distinguishing between the two and diagnosing the responsible drug.

## 1 Introduction

Endometrial cancer is one of the most common gynecologic malignancies, and its incidence has increased rapidly. Adenomyosis, a condition in which ectopic endometrial glands and stroma develop in the myometrium, can increase the risk of endometrial cancer development, similar to leiomyomas or polycystic ovary syndrome (1). Adenomyosis and endometrial cancer have similar traits: a local microenvironment that promotes the growth of endometrial stromal cells and an isoechoic area to the endometrial tissue around altered junctional zone as an ultrasound characteristic. These traits indicate the similarity in the pathophysiology of these conditions (1, 2). Several novel biomarkers, such as relative telomere length in cell-free DNA (3) and glandular cells in preoperative cervical smear (4), have been reported for the early diagnosis and management of endometrial cancer.

The traditional first line of treatment for endometrial cancer is platinum-based chemotherapy (5). However, treatment for advanced or recurrent endometrial cancer after platinum-based chemotherapy is not standardized. In December 2021, a combination of lenvatinib and pembrolizumab (LEAP) was approved in Japan for advanced endometrial cancer, based on the result of the KEYNOTE-775 clinical trial (6) and could be considered standard therapy for platinum-resistant advanced endometrial cancer.

In contrast to the limited antitumor effects of each drug individually, the combination of lenvatinib and pembrolizumab is more effective in advanced or recurrent endometrial cancer, regardless of the tumor’s genetic characteristics. Lenvatinib is a multiple tyrosine kinase inhibitor acting on vascular endothelial growth factor receptor (VEGFR), fibroblast growth factor receptor, platelet-derived growth factor receptor α, RET proto-oncogene, and KIT proto-oncogene. Lenvatinib has a limited efficacy against recurrent endometrial carcinoma when used as a single agent (7). Pembrolizumab is an inhibitor of programmed cell death 1, an immune checkpoint inhibitor. Antitumor effects of pembrolizumab have been reported in patients with microsatellite instability-high (MSI-H) or mismatch repair-deficient (dMMR) advanced endometrial carcinoma, while the effects are less significant in patients with microsatellite-stable or mismatch repair-proficient disease (8, 9).

A characteristic of this combination therapy, as opposed to traditional chemotherapy, is the variable adverse effects. The antitumor effects of traditional chemotherapy are mediated by cytotoxicity in malignant and benign cells. Contrarily, since molecularly targeted drugs are specifically toxic to malignant cells expressing the target protein, the adverse effects are also related to the target protein. Since immune checkpoint inhibitors mediate their antitumor effects by modulating the patient’s immune system, the associated adverse effects include autoimmune diseases. Medical professionals should be more aware of these adverse effects because several such adverse effects may be asymptomatic.

Posterior reversible encephalopathy syndrome (PRES) is characterized by neurological symptoms such as disturbance of consciousness, seizures, headache, visual disturbances, focal neurological deficit, and status epilepticus (10). It is thought to occur due to impaired autoregulation caused by damaged vascular endothelial cells.

PRES is one of the various adverse effects of lenvatinib. Because autoimmune encephalitis is also an adverse effect of pembrolizumab, it is difficult to diagnose PRES in patients with advanced endometrial cancer receiving a combination of lenvatinib and pembrolizumab. The similar phenotypes make it difficult to differentiate between these two conditions.

Herein, we report a case of advanced endometrial cancer with neurological manifestations of PRES caused by lenvatinib. The patient provided informed consent for the publication of this case report, including images.

## 2 Case description

The patient was a 53-year-old woman with no relevant medical history. She experienced discomfort and pain in the anal region, and a colonoscopy detected a tumor in the colon. On the basis of imaging and endometrial sampling cytology with conventional biopsy findings, she was diagnosed with International Federation of Gynecology and Obstetrics stage IVB endometrial cancer (endometrioid adenocarcinoma Grade 1) with colon metastasis and lymphadenopathy in the bilateral obturator lymph nodes and sacrum. She received neoadjuvant chemotherapy (four cycles of paclitaxel 175 mg/m^2^ and carboplatin area under curve 6). Two months later, Hartmann surgery was performed to prevent the tumor from occluding the colon. Pathological evaluation of the tumor specimen confirmed endometrial cancer, surgical stage IVB. MSI testing revealed the tumor was MSI-H.

After the surgery, computed tomography (CT) showed an enlarged recurrent tumor in the colon, with peritoneal dissemination and multiple metastases in the paraaortic lymph nodes. Hence, she was started on a combination of lenvatinib (20 mg, administered orally once daily) and pembrolizumab (200 mg, administered intravenously as a 30-minute infusion every 3 weeks). On day 11 after the LEAP therapy, she received 4 units of red blood cells due to a fall in her hemoglobin level to 7.3 g/dL. She was discharged on day 12. On day 15, she developed a gait disorder and tremors. Hypothyroidism (thyroid stimulating hormone [TSH] level: 5.350 ng/mL, free thyroxine 4 [FT4] level: 0.99 pg/mL, free thyroxine 3 [FT3] level: 2.08 pg/mL) was also detected on the same day on consultation with endocrinologists.

**Figure 1:**
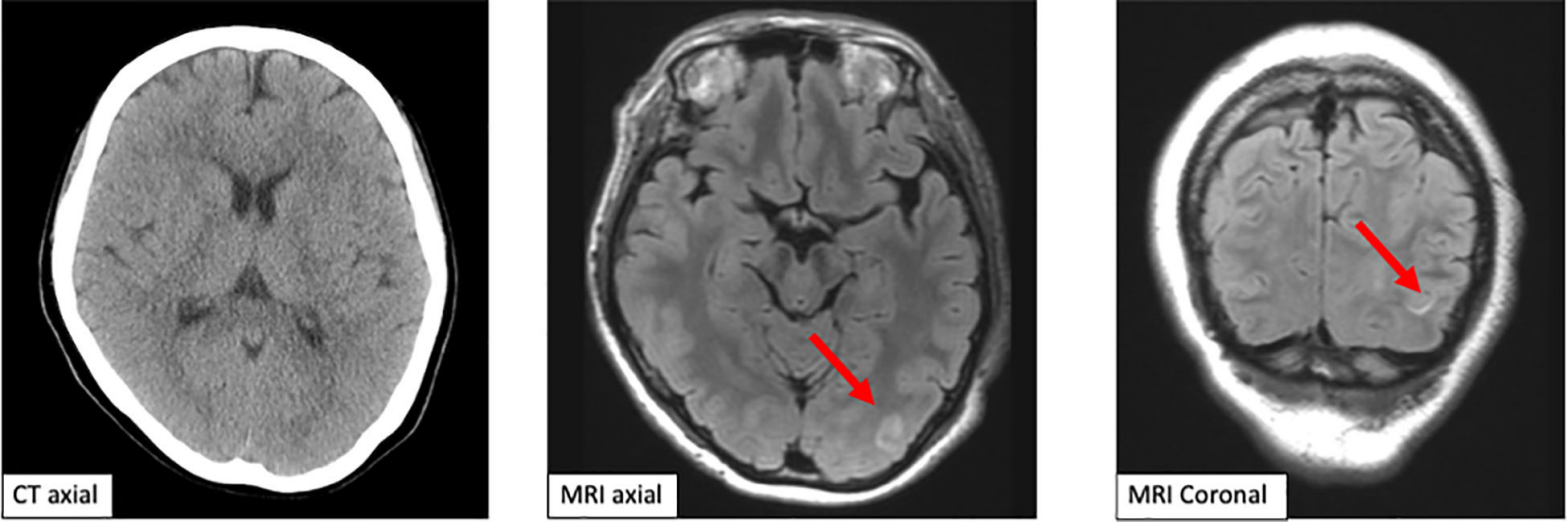
Computed tomography scan and magnetic resonance imaging of the brain. The emergent computed tomography scan of the brain shows no brain metastasis and intracranial hemorrhage. Magnetic resonance imaging shows a slightly high signal in the left occipital lobe axial and coronal.

**Figure 2:**
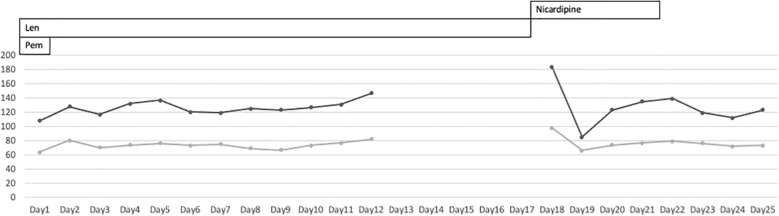
Patient’s blood pressure during lenvatinib-pembrolizumab therapy. The patient’s blood pressure was not monitored at home. It was abnormally high at the time of admission to the emergency room.

On day 18, she was referred to the emergency room for an altered sensorium. On arrival, her Glasgow Coma Scale score (**Supplementary Figure 1**) was E3V4M6. Her blood pressure showed a continued increase (**Figure 1**). There was no electrolyte imbalance or renal or liver failure (**Table 1**). An emergency CT scan found no brain metastasis or intracranial hemorrhage (**Figure 2**). Magnetic resonance imaging (MRI) showed a slightly high signal intensity in the left occipital lobe, with no apparent cerebral infarction (**Figure 2**). LEAP therapy was discontinued. Although there were no visual complaints or findings given the location of the MRI abnormalities and electroencephalogram was normal, her consciousness level gradually worsened, resulting in convulsions, which were suppressed by an intravenous injection of diazepam (5 mg). She was started on levetiracetam (200 mg) to prevent convulsions. For further investigation, additional blood tests and multiple lumbar taps were performed. While serum vitamin B1, TSH, FT4, and FT3 levels were normal, a slight increase was seen in the anti-thyroid peroxidase antibody levels (**Table 1**). The blood glucose level was 110 mg/dL. Analysis of the cerebrospinal fluid found cells (5/µL), protein (154 mg/dL), and glucose (50 mg/dL) (**Table 1**), suggesting that meningitis was unlikely. The disturbance in consciousness gradually improved with time, indicating the low probability of Hashimoto encephalopathy.

**Table 1:** Laboratory results on day 18.

Laboratory results		
**Blood test**	γ-GTP 36U/L	Cort 9.5ug/dL
**RBC 303 × 10^4^/µL**	**CK 215U/L**	**ACTH 6.3pg/mL**
**Hb 9.3 g/dL**	BUN 14.9mg/dL	TgAb 10.0IU/mL
**Plt 7.8 × 10^4^/µL**	Cr 0.68mg/dL	
**WBC 24 × 10^2^/µL**	**UA 5.8mg/dL**	**VBG**
PT% 100.0%	Na 144mEq/L	pH 7.403
INR 0.98	K 3.3 mEq/L	**PCO2 47.3mmHg**
APTT 31.6sec	Cl 104mEq/L	**PO2 48.4mmHg**
Fib 237mg/dL	Ca 9.2mg/dL	HCO3 28.8mEq/L
**D-dimer 5.3 ug/mL**	P 3.7mg/dL	**BE 3.5mEq/L**
TP 6.5g/dL	BS 78mg/dL	
Alb 3.1g/dL	NH3 22µg/dL	** CSF**
T-Bil 0.91mg/dL	**CRP 1.25mg/dL**	Cells 5/µL (Lym 5/µL)
D-Bil 0.15mg/dL	**TSH 7.970µIU/mL**	Protein 154mg/dL
**AST 67U/L**	FT4 0.96ng/dL	Glu 50mg/dL
**ALT 44U/L**	FT3 1.91 pg/mL	Cl 126Eq/L
**LD 384U/L**	VitB1 24ng/mL	ADA 3.6U/L
ALP 137U/L	HbA1c 5.5%	Viruses negative

Venous Blood Gas (VBG), Cerebrospinal Fluid (CSF). Laboratory test abnormalities are in bold.

Previous clinical trials have revealed that the incidence of adverse effects of lenvatinib and pembrolizumab on the central nervous system was 0.4% (11) and less than 0.1% (12), respectively, and could have caused PRES and encephalitis, respectively. The absence of markers of inflammation in the cerebrospinal fluid and a high signal intensity in the left occipital lobe on MRI suggested PRES, rather than encephalitis. Therefore, it was concluded that these symptoms were caused by lenvatinib, not pembrolizumab. She was resumed on treatment with pembrolizumab. Although no long-term sequalae of PRES were observed, unfortunately, CT showed multiple lymph node metastases after four cycles of pembrolizumab monotherapy, indicative of further disease progression. Pembrolizumab was discontinued, and she is now enrolled in another clinical trial in Japan.

## 3 Discussion

Patients with advanced or recurrent endometrial cancer could experience neurological symptoms for many reasons. Lenvatinib and pembrolizumab adversely affect the central nervous system and may cause PRES and encephalitis, respectively. Cancer-related thrombophilia can cause cerebral infarction, and associated brain metastases may result in hyperten sive intracranial hemorrhage. Identifying the cause of neurological symptoms is crucial, especially when it is drug related. If lenvatinib causes PRES in MSI-H/dMMR patients, effective therapy with pembrolizumab alone can be restarted after cessation of lenvatinib.

**Figure 3:**
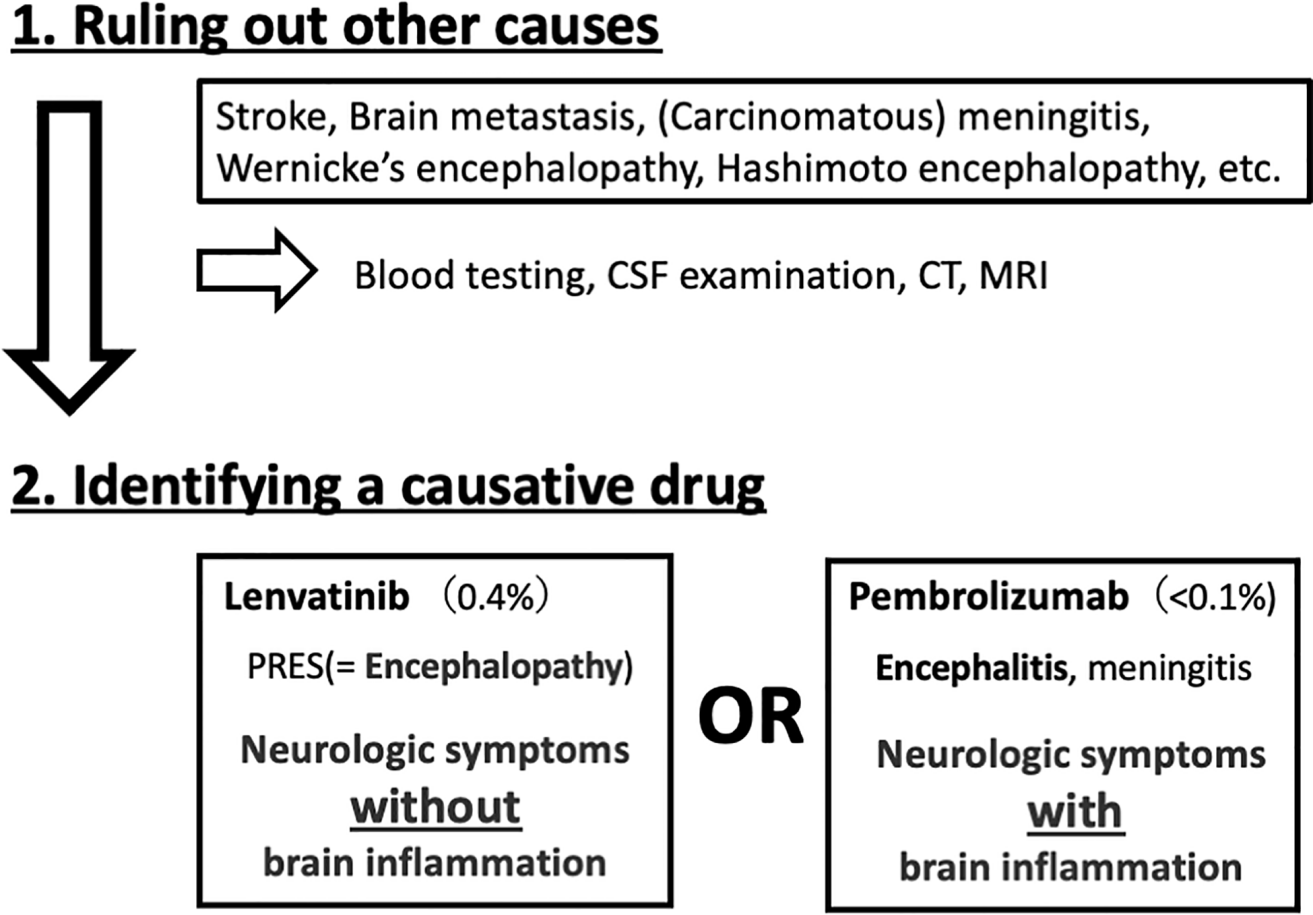
Steps to treat neurological adverse effects.

The difference between encephalopathy and encephalitis is key to distinguishing between lenvatinib- and pembrolizumab-related adverse effects. Although both present with neurologic symptoms, encephalitis is characterized by inflammation in the brain, while encephalopathy is non-inflammatory. A hyperimmune response due to high cytokine levels or cerebral edema due to vascular hyperpermeability can cause encephalopathy. A lumbar puncture is the best procedure to distinguish between these two conditions by confirming or ruling out brain inflammation (**Figure 3**).

PRES is characterized by interstitial edema caused by hyperperfusion resulting from a disruption in autoregulation due to damaged vascular endothelial cells. This damage is thought to be caused by hypertension, sepsis, pre-eclampsia, eclampsia, autoimmune disorders, and immunosuppressive or cytotoxic drugs (10). Cytokines, such as tumor necrosis factor-α, interleukin-1, and VEGF, increase vascular permeability. A common side effect of lenvatinib is hypertension. Lenvatinib-induced hypertension and VEGFR inhibition are closely associated with vascular endothelial cell damage. Pembrolizumab, when given in combination with lenvatinib, also acts as an immunomodulator and further increases vascular permeability. Hence, LEAP therapy increases the risk of PRES.

Brain imaging in PRES shows several distribution patterns, of which the holohemispheric watershed superior frontal sulcus and dominant parietal-occipital patterns are the most common. High signal intensity on T2-weighted/fluid-attenuated inversion recovery images and high apparent diffusion coefficient are seen, reflecting vasogenic edema. This patient showed a dominant parietal-occipital pattern (13).

Following the development of PRES, the LEAP therapy was suspended, and pembrolizumab monotherapy was instituted. The limitation of this case report is that the patient was fully informed about the side effects of the treatment on day 12 and was given discharge instructions, including home blood pressure monitoring. However, blood pressure control at home was inadequate, and the patient’s physical condition was unknown until the emergency department visit on day 15. To the best of our knowledge, this is the first report of resuming chemotherapy after a differential diagnosis of urgent intervention-indicated encephalopathy. Our findings highlight that a thorough causal inference is critical for continuing effective therapy.

## Data availability statement

The original contributions presented in the study are included in the article/**Supplementary Material**. Further inquiries can be directed to the corresponding author.

## Ethics statement

Written informed consent was obtained from the patient to publish any potentially identifiable images or data included in this article. The study was approved by the ethics committee of the medical faculty at Teikyo University Hospital.

## Author contributions

All authors contributed to the article and approved the submitted version. YM designed the report. YM and KN wrote the manuscript. YM and KN collected the patient’s clinical data. YM, HN, TK, KS, KT, TI, MH, HH, and KN was responsible for the conception and revision of the manuscript. YM, HN, TK, KS, KT, TI, and KN carried out the patient management. All authors contributed to the article and approved the submitted version. This research received no external funding.
