# Supplementary material for: Case report: Posterior reversible encephalopathy syndrome, an adverse effect of lenvatinib and pembrolizumab combination therapy, in a patient with advanced endometrial cancer
Source: Front Oncol. 2023 Jan 20;12:1079716. doi: 10.3389/fonc.2022.1079716 (PMC9895820; doi:10.3389/fonc.2022.1079716)
Supplement: Supplementary file 1 [file DataSheet_1.pdf]

GLASGOW COMA SCALE : Do it this way

GCS EYES VERBAL MOTOR

Institute of Neurological Sciences NHS Greater Glasgow and Clyde

CHECK

For factors Interfering with communication,
ability to respond and other injuries

OBSERVE

Eye opening , content of speech and movements of right and left sides

STIMULATE

Sound: spoken or shouted request
Physical: Pressure on finger tip, trapezius or supraorbital notch

RATE

Assign according to highest response observed

Eye opening

Criterion	Observed	Rating	Score
Open before stimulus	✓	Spontaneous	4
After spoken or shouted request	✓	To sound	3
After finger tip stimulus	✓	To pressure	2
No opening at any time, no interfering factor	✓	None	1
Closed by local factor	✓	Non testable	NT

Verbal response

Criterion	Observed	Rating	Score
Correctly gives name, place and date	✓	Orientated	5
Not orientated but communication coherently	✓	Confused	4
Intelligible single words	✓	Words	3
Only moans / groans	✓	Sounds	2
No audible response, no interfering factor	✓	None	1
Factor interferring with communication	✓	Non testable	NT

Best motor response

Criterion	Observed	Rating	Score
Obey 2-part request	✓	Obeys commands	6
Brings hand above clavicle to stimulus on head neck	✓	Localising	5
Bends arm at elbow rapidly but features not predominantly abnormal	✓	Normal flexion	4
Bends arm at elbow, features clearly predominantly abnormal	✓	Abnormal flexion	3
Extends arm at elbow	✓	Extension	2
No movement in arms / legs, no interfering factor	✓	None	1
Paralysed or other limiting factor	✓	Non testable	NT

Sites For Physical Stimulation

Finger tip pressure

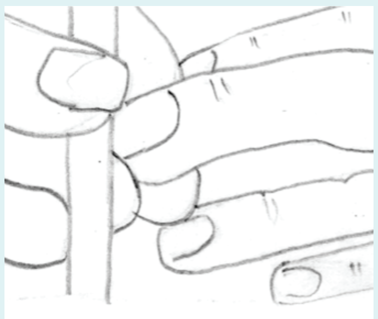

Trapezius Pinch

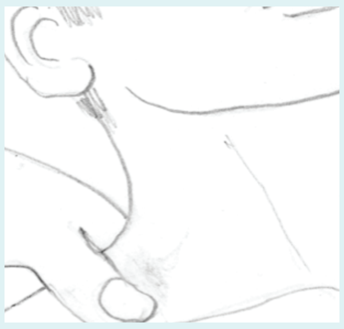

Supraorbital notch

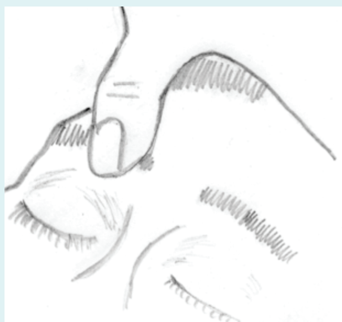

Features of Flexion Responses

Modified with permission from Van Der Naalt 2004
Ned Tijdschr Geneeskd

Abnormal Flexion

Slow Sterotyped
Arm across chest
Forearm rotates
Thumb clenched
Leg extends

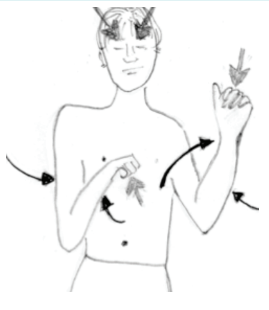

Normal flexion

Rapid
Variable
Arm away from body

For further information and video demonstration visit www.glasgowcomascale.org

Graphic design by Margaret Frej based on layout and illustrations from Medical Illustration M I • 268093

(c) Sir Graham Teasdale 2015
